# Equality, equity, diversity, and inclusion principles: how should we apply these to statistical methodology research?

**DOI:** 10.1186/s12874-025-02564-8

**Published:** 2025-04-24

**Authors:** Ellesha Smith, Carol Akroyd, Rebecca Barnes, Emma Beeston, Jonathan Broomfield, Sylwia Bujkiewicz, Natalie Darko, Christopher Newby, Mark J. Rutherford, Aiden Smith, Rachael Stannard, Freya Tyrer, Laura J. Gray

**Affiliations:** 1 Department of Population Health Sciences, University of Leicester, Leicester, UK; 2 NIHR Applied Research Collaboration East Midlands, Leicester, UK; 3 NIHR Research Support Service (RSS) Hub, delivered by the University of Leicester and Partners, University of Leicester, Leicester, UK; 4 NIHR Biomedical Research Centre Leicester, University of Leicester, Leicester, UK; 5 Medical School, University of Nottingham, Nottingham, UK

**Keywords:** Equality, Diversity, Inclusion, Equity, Toolkit, Statistical methodology

## Abstract

**Background:**

The aim of equality, equity, diversity, and inclusion (EDI) is to ensure fair treatment, equal opportunities, equitable outcomes, and representation. The NIHR Research Design Service (RDS) EDI toolkit (https://www.rssleicesterresources.org.uk/edi-toolkit) helps researchers embed EDI throughout their work. This study evaluated the applicability of the RDS EDI toolkit for statistical methodology research and proposed adaptations to enable statistical methodologists to embed EDI in their research.

**Methods:**

A full-day meeting was held to consider how the RDS EDI toolkit could inform the inclusion of EDI principles in statistical methodology research. Twelve individuals attended from the University of Leicester and the NIHR Research Support Service (RSS) Hub delivered by the University of Leicester and Partners. At the meeting, definitions of statistical methodology research and EDI were agreed. The RDS EDI Toolkit was interrogated to identify relevant aspects and additional considerations for statistical methodology research.

**Results:**

Overall, the RDS EDI toolkit was valuable for incorporating EDI in statistical methodology research. Five recommendations to supplement the toolkit are proposed to reflect specific EDI challenges for statistical methodology research. Statistical methodology researchers should:

1. Perform formal assessments of the required resources for maximising EDI from the outset of statistical methodology research projects, including consideration of research team, training, patient and public involvement, and appropriate budgeting.
2. Conduct prospective and retrospective context-specific evaluations of the impact of their methodological research on exacerbating or reducing inequalities.
3. Evaluate the selection of data sets, work with multiple, diverse databases, or use data sets that have undergone equality impact assessments.
4. Clearly communicate EDI assessments and limitations, including which data sets are used and their purpose, such as illustrating or comparing methods, informing simulations, or guiding clinical practice.
5. Incorporate EDI in dissemination activities and advocate for EDI principles in the peer review process.

**Conclusions:**

Embedding EDI principles throughout statistical methodology research will improve its relevance and quality, better serve the public, and build public trust. It is essential that statistical methodologists strive towards equity in all aspects of their work. This paper demonstrates the value of the NIHR RDS EDI toolkit for statistical methodology research and encourages methodologists to adopt the recommendations in this paper. Further extensions to this work are needed to seek the wider views and experiences of statistical methodologists and public contributors from diverse and under-represented groups.

## Introduction

Equality, equity, diversity, and inclusion (EDI) represent core values centred around the fairness of opportunity. Under the UK Equality Act (2010) [34], discrimination against individuals’ protected characteristics, including race, religion and belief, age, sex, disability, sexual orientation, gender reassignment, pregnancy and maternity, and marriage and civil partnership, is prohibited. Equality refers to treating all people the same (e.g., offering the same opportunities and equal access to resources), while equity refers to achieving fair outcomes, recognising diversity, and addressing inequality by giving people what they need to achieve their potential [22]. Diversity relates to the recognition and respect of differences, including the involvement of people in wider communities. Inclusion refers to welcoming and valuing individuals from all backgrounds. Taken together, EDI aims to ensure fair treatment and opportunities for all, by removing “prejudice and discrimination on the basis of an individual or group of individuals'protected characteristics” [36].

This article relates to EDI for statistical methodology research with future applications to health and social care research. Statistical methodology research focuses on developing mathematical formulae, models, techniques, and software to analyse research data and design research studies effectively. Statistical methodology research encompasses a range of approaches from the more applied end of the spectrum – such as the comparison of existing methods for the analysis of clinical trial data – to the more technical end comprising model developments or extensions, such as introducing a new bias correction method for maximum likelihood estimators [11].

In the UK, EDI is central to most research funding schemes. For example, in 2022, the UK’s National Institute for Health and Care Research (NIHR) published its first EDI Strategy outlining its commitment to EDI [22]. UK Research and Innovation (UKRI) and Wellcome have also set out their EDI visions [35, 39]. Given that statistical methodology research is funded through these schemes, and considering the benefits of embedding EDI principles, such as improving the relevance and quality of research, fostering innovation and problem-solving, better serving the public, and building public trust, statistical methodologists must begin to consider integrating EDI into their work.

Parallel to UK efforts, EDI in health research is also a global priority, with many countries outside of the UK implementing EDI initiatives. However, while this paper uses the UK terminology ‘equity, equality, diversity and inclusion’, similar concepts are termed differently internationally. In the United States, the Association of American Medical Colleges publishes resources and initiatives aimed at promoting diversity, equity, inclusion, and accessibility (DEIA) in biomedical research [3]. Canada’s Tri-Agency Equity, Diversity and Inclusion Action Plan focuses on improving equitable and inclusive access to funding opportunities, including supporting Indigenous research and training [4]. Similarly, the European Union integrates EDI into its Horizon Europe framework by mandating Gender Equality Plans for funding from 2022 [6].

For applied healthcare research, several tools have been developed to support researchers in embedding EDI principles and impact assessments into their work, such as the Wellcome Trust’s Act Boldly toolkit [16], the Association of Medical Research Charities (AMRC) Equity, Diversity and Inclusion Resource Hub (https://www.amrc.org.uk/pages/category/edi-resource-hub), the FOR EQUITY Health Inequalities Toolkit (https://forequity.uk/), and NIHR Applied Research Collaboration East Midlands Equality Impact Assessment (EqI) toolkit (https://arc-em.nihr.ac.uk/arc-store-resources/equality-impact-assessment-eqia-toolkit). However, none of the existing toolkits focus on the unique challenges associated with statistical methodology research.

The NIHR Research Design Service (RDS) EDI toolkit was developed by NIHR RDS East Midlands in 2022 to inform applied health and social care research studies. It was developed with valuable and broad input from the RDS EDI Group and other RDS and NIHR colleagues, researchers and public contributors. The toolkit was piloted in four RDS regions (East Midlands, North West, South East and West Midlands). RDS colleagues shared their views and helpful suggestions for improvement in focus groups, before it went live in 2022. Understanding how its principles and questions apply to statistical methodology research is essential to extend its relevance and applicability.

The toolkit (summarised in Table 1) aims to ensure that EDI is considered and encapsulated throughout research and consists of eight domains in specific areas. Each domain includes questions that prompt researchers to consider EDI throughout their research.

**Table 1:** Summary of the NIHR Research Design Service (RDS) EDI toolkit

**1. Historical context and structural inequalities:** Explore the roots of health inequalities, emphasising how structural inequalities, including systemic racism, shapes health and care research.
1. Has previous research excluded certain populations?
2. What historic and structural issues might affect how under-represented groups feel about research participation or involvement?
3. How will you navigate these issues?
**2. Research team:** Focuses on fostering diverse and inclusive research teams, including equitable opportunities for under-represented groups.
1. How diverse is your research team?
2. How culturally competent is your research team?
3. What difference has incorporating diverse perspectives and lived experiences made to your research design?
**3. Public involvement:** Provides strategies for involving diverse public stakeholders in a meaningful and accessible way.
1. How aware are you of equality, diversity and inclusion issues in public involvement?
2. How will your public involvement strategy enable you to recruit and retain diverse public contributors?
3. How will you support and empower your diverse public involvement group?
**4. Selection of participants, sites, and samples:** Provides guidance on recruiting diverse participants and selecting sites to ensure representation of under-represented groups.
1. How generalisable is your sample?
2. Has geographical need informed your choice of site(s)?
3. Are you excluding anyone, if so, is this justified?
**5. Data collection:** Recommends inclusive and culturally sensitive approaches to data collection.
1. How equitable is your data collection strategy?
2. Do your methods address participants’ protected characteristics, circumstances and needs?
3. What additional steps will you take to be inclusive of ‘seldom heard’ or marginalised groups?
**6. Data analysis and presentation:** Encourages researchers to analyse data in a way that reflects differences across diverse groups and avoids biased interpretation of results.
1. How will you analyse and report equality, diversity, and inclusion characteristics?
2. Have diverse perspectives been incorporated to support the interpretation of results?
3. How will you describe participants’ equality, diversity, and inclusion characteristics?
**7. Dissemination, implementation, and impact:** Advises tailoring of research outputs for diverse audiences, ensuring accessibility, and reaching under-represented groups.
1. Will findings be disseminated via accessible and inclusive formats and channels?
2. How will this research be taken forward to benefit all patients/service users across all settings?
3. Will the most in need be impacted by your research?
**8. Budgeting for inclusion:** Focuses on allocating resources to enable inclusive research.
1. What additional costs need to be factored in when designing inclusive research?
2. How might embedding equality, diversity and inclusion extend the timelines of your research?
3. Are there steps that researchers can take to reduce costs?

## Aims and objectives

Statistical methodologists should strive towards equity in all aspects of their work. However, currently there are no EDI toolkits or guidelines that specifically focus on the unique challenges of incorporating EDI in statistical methodology research. This study aims to address this gap by adapting and extending the toolkit to provide meaningful inclusion of EDI principles. Understanding how EDI principles apply to statistical methodology research is essential to improve its relevance and quality, better serve the public, and build public trust. Additionally, this study aspires to raise awareness of EDI issues and gaps in the guidance in the context of statistical methodology research.

A face-to-face meeting of EDI experts and statistical methodologists was held as an initial step towards achieving the above aims. The meeting focused on evaluating the application of the NIHR RDS EDI toolkit for statistical methodology research. We chose to focus on the NIHR RDS EDI Toolkit due to its alignment with funding frameworks and the ability to collaborate with the developers of the toolkit on the adaptions. The objectives of this study were to:

1. Evaluate the relevance of the existing NIHR RDS EDI toolkit for statistical methodology research.
2. Suggest recommendations for its adaption in the statistical methodology research context.
3. Provide a narrative discussion of EDI issues and gaps in the guidance related to statistical methodology research, incorporating real-life experiences and examples in the literature, to raise awareness and stimulate future discussion.

The study adopted a pragmatic lens to evaluate the toolkit's practical application and propose actionable recommendations to guide future statistical methodology research.

## Methods

Our methodology was informed by the EQUATOR Network toolkit for developing reporting guidelines [33]. Specifically, we followed the stages of guideline development in the toolkit, including identifying the need for a new guideline, generating a list of items for discussion and deciding meeting details, holding a face-to-face meeting, and reviewing the first draft of the guideline. Although we did not intend to develop a formal set of guidelines, there is some overlap in intended outcomes [33]. Ethical approval from the University of Leicester (reference: 43552) was granted prior to the start of this work.

### Participants

We identified prospective participants locally, focusing on people with relevant expertise in statistical methodology or EDI, and ensuring representation across a range of career stages, from early career researchers to senior management. We recognise that these definitions may differ in interpretation internationally, so for the purpose of this study, we define the following career stages:

• PhD/early career researcher: Individuals in the first five years of their research career, including PhD students.
• Post-doctoral researcher: Individuals who have completed their PhD or have more than 5 years research experience and are employed in research roles, such as research fellows.
• Academic: Individuals holding tenured positions within a university, who have significant decision-making responsibilities within the research process, including lecturers and professors.
• Senior management: Individuals with formal management responsibilities within a university or research institution, such as leading a research group or head of departments, and are responsible for overseeing research strategy, resources and staff.

Our aim in this study was to capture a broad range of perspectives and expertise to ensure that recommendations reflect the requirements and experiences of researchers across all career stages. We purposively sampled 13 individuals from the University of Leicester and the NIHR Research Support Service (RSS) Hub, delivered by the University of Leicester and Partners. We included EDI leads (including authors of the RDS EDI toolkit and representation from the Centre for Ethnic Health Research), those holding departmental EDI roles (such as EDI Champion or Athena Swan lead), and statistical methodologists. We recognise the importance of diversity and collected information on protected characteristics, career stage and expertise of the group. However, due to the small numbers and to maintain confidentiality of EDI data, we report the demographics qualitatively to provide transparency about the composition of the group. Informed consent was obtained from all participants.

### Facilitated meeting

Informed by the EQUATOR toolkit for developing reporting guidelines, a full-day face-to-face consensus meeting was held to consider how the RDS EDI toolkit could inform statistical methodology research. Prior to the meeting, participants were asked to read through the NIHR RDS EDI Toolkit and consider the relevance of each domain for statistical methodology research to discuss at the meeting. The day was externally facilitated by Nifty Fox Creative who also live-scribed the event. Live-scribing is the real time illustration of the verbal discussions in pictures and text; it produces useful aids for engaging the participants and facilitating the discussion effectively. These visual records also supported the writing up of the discussion and recommendations presented in this paper. The facilitation ensured we achieved the objectives of the day and completed all required tasks. Over the day we:

1. Set out agreed definitions of statistical methodology research and EDI and began to consider how they intersect;
2. Interrogated the existing RDS EDI Toolkit by domain to consider:
  - What within the domain is relevant as is to statistical methodology research?
  - What within the domain could be relevant if adapted to statistical methodology research?
  - What within the domain could be relevant if more research is undertaken in statistical methodology research?
3. We then considered what is missing from the current toolkit and considered:
  - What is unique to statistical methodology research?
  - What could be added to the toolkit that already exists?
  - What we don’t yet know/what research needs to be done?
4. We then focussed on two priority issues and put together an action plan.

During the meeting, participants shared examples from their own experiences and from the literature to elaborate on the applicability of the toolkit in statistical methodology research. The meeting was not recorded verbatim to ensure participants felt comfortable in open discussion; however, the facilitator recorded the themes and live-scribed notes of the discussion. Following the meeting, two researchers led the write-up of this paper, using the live-scribed notes from the meeting, as the basis for the Results section. The Results section was structured domain-by-domain to reflect the structure of the meeting and present a narrative discussion of the themes and insights drawn from participants’ shared experiences, examples from the literature, and reflections on the relevance and applicability of the toolkit for statistical methodology research. After drafting the initial results, the write-up was sent to all participants to review, allowing them to contribute additional thoughts or experiences after reflecting on the meeting and the write-up.

### Researcher positioning and reflexivity statement

Thirteen participants were recruited. Of these, 12 attended the facilitated meeting but all have contributed to ongoing discussions and the drafting of this paper; therefore, all of the participants are authors of this paper. The authors bring diverse perspectives shaped by intersections of gender, ethnicity, neurodiversity, age and other aspects of identity. Some authors identify as being from underserved groups and specific protected characteristics vary within the group. However, we acknowledge that we may not be fully representative of wider populations. Our professional backgrounds include clinical design, evidence synthesis, epidemiology, machine learning and our collective experience spans academic, clinical and applied research contexts.

Acting as both researchers and participants in the study, we recognise that the dual roles played in this study introduced the potential for bias. To mitigate this, we employed structured discussion, live-scribing, and an external facilitator.

### Patient and Public Involvement (PPI)

After discussions with the external facilitator, it was decided not to include public participants at this stage. The end-users of the updated toolkit with the recommendations in this paper are statistical methodologists, rather than public members, so providing the perspectives of a broader range of statistical methodologists was prioritised. However, we acknowledge that this research reflects interpretations shaped by our own perspectives and recognise the value of incorporating broader perspectives; future plans for achieving this are outlined in the Discussion section.

PPI members who provided input on the toolkit for applied health researchers said that they needed an adapted version. A resource for PPI members to use parallel to the toolkit is currently under development by the NIHR Research Support Service (RSS) Hub delivered by the University of Leicester and Partners.

Alongside this project we have been undertaking a programme of research focusing on how to undertake impactful PPI for statistical methodology research (https://leicesterbrc.nihr.ac.uk/ppismart/), demonstrating our commitment to this important topic. The findings from this work were fed into our considerations of the public involvement and budgeting for inclusion sections.

### Phases of methodological research in biostatistics

There is a blurred boundary between statistical methodology research and applied research conducted by a statistician. For example, the development of a prognostic model may contain methodological elements – such as the comparison of different modelling approaches. The framework for methodological development in biostatistics, proposed by Heinze et al. [11], is useful for clarifying what type of statistical methodology research is being referred to in this paper. This framework includes four phases of methodological research: phase 1 introduces a new idea, demonstrates its validity by investigating its properties, and includes either no or very simple example data analyses. Phase 2 demonstrates the use of the method with real data but in only a limited range of applications. Phase 3 compares the new method with competitors and demonstrates its use in a wide range of applications. Phase 4 summarises the evidence about a method, uncovering previously unknown behaviour of the method in complex data analyses, and in an extended range of applications. Simulation studies are implemented through Phases 2 to 4 to investigate different aspects or properties of methods. These phases are referred to throughout the results section in this paper to highlight how different domains apply to different statistical methodology research phases.

## Results

### Adapting the toolkit for statistical methodology research

The results of this study are presented by domain to reflect the structure of the face-to-face meeting. Themes and insights discussed during the meeting were drawn from participants shared experiences, examples from the literature, and reflections on the relevance and applicability of the toolkit for statistical methodology research. The following sections provide a detailed narrative account of the findings from the meeting across each domain.

#### Historical context and structural inequalities

A unique difference between most statistical methodology research and applied health and social care research is that, usually, we do not collect new data. Typically, existing data sets (either from completed research projects, cohorts or routinely collected data) are used or artificial data is simulated, which may or may not be based on an existing data set. Data simulation involves creating (often simplified) synthetic data sets from known distributions and parameters (which can also be informed by existing data) such that the underlying truth of the data generation is known. This is ideal for understanding if new statistical methods perform as expected, and under which range of scenarios performance is satisfactory.

We hypothesise that there might be instances where using data that are unrepresentative of protected characteristics could lead to recommendations around particular statistical approaches which, when used within future applied studies, may exacerbate health inequalities. For example, the Framingham Risk Score, a cardiovascular disease risk prediction model, was developed on a data set of mostly white Americans. Applying unrepresentative models in clinical practice can lead to inaccurate risk assessments, potentially worsening health inequalities for underrepresented populations [32]. Further research on this topic is required to establish the potential scale of this issue and to gain consensus over the motivation for equity assessments when using these types of methods [38]. However, for phases 2 to 4 in the methodological research framework, the choice of the data set and the representativeness of this data is of crucial importance, given the results of such models can affect clinical decisions.

For applied health and social care studies, equality impact assessments were developed for researchers by NIHR Applied Research Collaboration East Midlands and a wider audience by Public Health England [23, 27]. The NIHR Biomedical Research Centre REP-EQUITY group also developed a toolkit for investigators to consider in facilitating representative and equitable sample selection [29]. These assessments are used at the planning stage to ensure that policies, practices, and activities will not have an unintended discriminatory impact on people with protected characteristics. We recommend that future research looks to develop equality impact assessments to evaluate existing data sets for methodological research. Undertaking such an assessment would highlight potential areas of concern regarding representativeness. Based on these, researchers may decide to replace data with a more representative data set, add additional data sources, or ensure that such limitations and the potential implications of these are included in any output from their research.

Regarding how structural inequalities and historic events might affect how underserved groups feel about research participation or involvement, we recognise that there may be groups of individuals who have a distrust of data or statistics, and who may be less likely to participate in research or PPI activities. Groups that have been previously exploited by researchers or excluded from research activities often have distrust in the research community and the healthcare system generally. For example, the Pima Indian Diabetes Database (PIDD), which originated from an observational study beginning in the 1980s, was made open source without the consent of the Akimel O’odham people who participated in the study. The data set is often used in machine learning research as an example data set. To this day there are frequently published papers that present small variations in supervised machine learning algorithms that demonstrate incremental improvements in model performance, rather than improving the lives of the people in this community. This historical exploitation has led to the people in this community being distrustful of researchers and they have since refused to be a part of any research studies [28]. The historical context of data sets used in research and the impact of doing so on the original participants should be considered, even for statistical methodological papers where the application is illustrative and not the main objective of the research.

In summary, this domain is highly relevant to all research, including statistical methodology research. There are nuances to this type of research that may need consideration, such as the representativeness of existing data sources and barriers to participation or PPI. There is a need for additional research to establish how failing to account for EDI principles can lead to applying methods that adversely disadvantage some population groups and how to appraise existing data sets according to EDI principles.

#### Research team

This domain is relevant to all research areas including statistical methodology research. It is important and beneficial for research teams in every field of study to be diverse and provide unique perspectives. One distinction of this domain for statistical methodology research discussed by the statistical methodologists at the meeting is that researchers in this discipline often work independently or within small teams. In addition, as discussed previously, the data are usually previously obtained, or an example data set is used for methodological development, which means that fewer researchers are required for statistical methodology projects. The nature of this type of research may be perceived to be not conducive to ensuring that research teams are diverse but it is essential that methodological researchers consider involving more researchers with diverse characteristics. Similar to how PPI can provide important perspectives for methodology development, interdisciplinary collaboration could also increase the diversity of research teams and provide unforeseen and innovative insights. For example, receiver operating curves that are used in medical statistics to evaluate the diagnostic accuracy of medical tests were originally developed from signal detection theory to improve the accuracy of radar detection during World War II [14], while qualitative methods may also provide unique insights into statistical methodology research. Practical recommendations to guide interdisciplinarity, including rotating roles, training, and EDI monitoring audits, have been recently published [37].

Statistical methodology research is a specialised and technical field of study, requiring higher level education, which may attract a narrower group of individuals to these roles that lack diversity. Training and reflection on EDI principles within statistical methodology research groups and for recruitment to research teams can be undertaken, which may have been historically overlooked. Encouraging students from diverse backgrounds to develop expertise and pursue careers in statistical methodology research from early on in their secondary education is important for overcoming career pipeline issues. For example, outreach to secondary schools, that is accessible and inclusive of minoritised groups, provides statistical and scientific exposure that students may not otherwise receive [15]. In health professions generally, evidence suggests that diversity can be improved through the university admissions process [7]. Additionally, there are undergraduate internships aimed at students from minoritised groups, such as the Health Data Research (HDR) UK Health Data Science Black Internship programme [10]. The Medical Research Council (MRC) EDI Strategy Initiative: Black in Biomedical Research project also uses a similar approach to increase representation of researchers from Black heritage backgrounds across the MRC and the biomedical sciences [20]. Within statistics, there is evidence that workshops and mentoring programmes have the potential to attract and retain women in the field [19]. Outreach in schools and internship opportunities can improve the lack of diversity in methodological research by demonstrating diverse role models from wide-ranging backgrounds and addressing stereotypes, such as the misconception that only ‘gifted’ individuals should pursue careers in technical fields like statistical methodology research [30].

#### Public involvement

We deemed this domain highly relevant to all types of research, including statistical methodology research. Recommendations in the toolkit, including making meetings accessible and inclusive, being aware of power dynamics, considering community venues for PPI meetings, and covering the costs of childcare or respite care, are all useful suggestions for statistical methodology researchers to be aware of when planning PPI. However, the nature of statistical methodology research means that there are additional considerations to achieve diverse involvement. For example, there is less public awareness of this type of research so it might be more challenging to ensure diversity as opportunities to be involved in statistical methodology research may appeal to some groups more than others. For example, those who have undertaken higher education in numerical disciplines or have had careers in sectors requiring such skills may be more likely to express an interest in such opportunities. Therefore, accessible promotion of involvement opportunities and highlighting the impact on patients or health services is crucial to achieving diversity.

The University of Leicester PPI-SMART group is an excellent demonstration of how more research and information and the further development of guidance and resources are essential to raise awareness and improve the quality of PPI in statistical methodology research. A recent survey by the group assessing current practices and attitudes of researchers in incorporating PPI in statistical methodology research found that only 21% of researchers had undertaken PPI during a methodological project but would incorporate meaningful PPI if there were more resources and guidelines [1]. Training in undertaking PPI and communication skills may help increase confidence and shadowing PPI activities for other types of research may also be of benefit. The PPI-SMART group continues to raise awareness of this issue through workshops and has started a shadowing scheme to increase researcher confidence. They have also developed resources for statistical methodologists, including case studies, animations describing statistical methodology research and explaining why PPI in this area is important, and a statistical glossary. The animation can enhance future PPI activities, increase researcher confidence and support recruiting public contributors [1], [40]. In addition, the group continues to undertake public engagement activities in community settings to raise awareness of statistical methodology research, which may help recruit from under-represented communities.

#### Selection of participants, sites and samples

As previously discussed, statistical methodologists often use simulated or historical data and are not involved in participant recruitment or data collection, so this domain has similar issues for statistical methodologists to those discussed in the Historical and Structural Issues domain. The purpose of the data used in methodological projects is to demonstrate the method, so often the generalisability of the participants is not of particular concern. This especially applies in the first and second phases of methodological development which use no or limited data for proposing the novel methodology. However, although often overlooked, this domain is relevant to the later phases of methodological development research that apply methods to real-life data. Sometimes, in phases 3 to 4, the results of the methodological application are not of primary importance, and methodological choices (e.g., the handling of missing data, simulating from a real-life data set) are made to simplify the application, which would be inappropriate for clinical interpretation. Although the participant characteristics are of secondary importance for demonstrating a method, statistical methodologists should be aware of and assess the future impact of naive application.

A recommendation for statistical methodologists is to consider this domain of the toolkit to be the selection of data sets, rather than participants, sites, and samples, and to work with multiple, diverse databases or use data sets that have undergone equality impact assessments. It should also be reported when the purpose of applications is purely methodology demonstration and EDI has not been considered to prevent the future inappropriate interpretation of the results.

#### Data collection

As noted above, most statistical methodology research will not require the recruitment of participants to a research study, hence the previous recommendations will also apply here.

There are some exceptions to this. There may be studies that take a qualitative approach to answer a statistical methodology question, such as the interview study by Kristunas [18], which aimed to understand feasibility issues in stepped wedge trials by interviewing people from a range of clinical trials related professions [18]. Consensus methods are also used within statistical methodology research, such as Delphi studies. For example, one study developed a framework for defining and interpreting the use of surrogate endpoints in interventional trials [5]. The setting of prior distributions within a Bayesian framework may also use elicitation methods, requiring the input of experts and/or public contributors [31]. Although these studies tend to recruit ‘professional’ participants, the same EDI considerations should be taken. In terms of diversity, researchers can ‘equality monitor’ by collecting demographics including geography, type of institution, career stage, socioeconomic status, as well as protected characteristics.

Researchers should evaluate how they can conduct their research to improve the inclusivity of research practices and enable contributions from diverse perspectives. This assessment will be specific to each research project but examples might include providing the option for hybrid or online participation, adapting the timings of research activities for people with childcare commitments or part-time researchers. Other practical steps might also include offering closed captions or alternative language options, ensuring websites are screen-reader compatible or designing resources that are accessible for individuals with colour vision deficiency. To address disparities, researchers might find ways to accommodate participants from under-resourced institutions or at different career stages where costs and resources may be barriers to participation. Additionally, it may be appropriate to recognise and compensate professionals from minoritised groups for their expertise to ensure that those professionals do not continue to carry the inequitable burden of educating the majority.

#### Data analysis and presentation

For statistical methodology development, the incorporation of EDI principles in data analysis and presentation is context-specific. For example, for research in the first phase of the methodological development framework where a new method is proposed theoretically, without application to a data set, there are no characteristics to report on. However, if methodology researchers took an EDI lens to their research, the research questions would also change and, in turn, the methodological developments would reflect this. An EDI approach to methodological research could potentially impact the statistical methodology that is developed because we *should* stratify or quantify differences across groups.

Additionally, if a method is developed based on issues discovered in real-world data set applications, it is important to assess how the data affected the development. One example of this is in evidence synthesis. It has historically been standard practice to explore heterogeneity in evidence synthesis by including average covariate values in standard meta-regression of study-level data. However, exploration and development of individual participant data meta-analysis demonstrated the flaw of this methodological approach in detecting covariate-treatment interactions: aggregation bias. Aggregation bias, or ecological bias, can exacerbate inequalities. For example, if there are differences in treatment effects across ethnicities, a standard meta-regression could show no difference due to study-level confounding. An example of aggregation bias that is often used to illustrate the concept is an ecological study that showed a strong correlation between average fat consumption and breast cancer mortality [26]. However, this result demonstrated a limitation of ecological studies: confounding by other correlated variables. Several risk factors for breast cancer, such as late age at first birth, low parity, and postmenopausal obesity, are higher in developed countries with higher fat intake, therefore predicting a high incidence of breast cancer [2, 12, 25]. In this example, the choice of methodology was not appropriate and led to misleading conclusions. Comparatively, individual participant data meta-analysis can estimate both study-level and individual-level interaction effects. This method was later developed as access to individual participant data became more available to statistical methodology researchers and they became aware of aggregation bias.

The rapid development of machine learning and artificial intelligence presents challenges to EDI, particularly with the rise of big data and increasing accessibility of tools to wider audiences with limited knowledge of EDI principles or statistical methodology. There is potential that biases embedded in these technologies could exacerbate existing inequalities [13, 24]. This highlights the importance of prioritising robust, inclusive and interpretable research methodology to ensure equitable outcomes as these technologies continue to evolve.

In the later phases of the methodological research framework, where data sets are used to illustrate methods in a variety of applications, it would be possible to report and consider the effect of EDI characteristics. The third and fourth phases would provide a good opportunity in methodological development for researchers to consider the impact on EDI principles if the methods were implemented in the future by evaluating and testing the methods with a variety of EDI-related characteristics. Many standard applications of statistical methods will disadvantage under-represented groups if there are differences in outcomes. For example, an average treatment effect will be driven by those in the majority groups in a study. Average treatment effects are usually reported in randomised controlled trials and observational studies of comparative effectiveness. However, the same treatment can have varying effects in different people with varying prognostic characteristics. If only the average treatment effect is reported, the difference in effect for minority groups would be missed, leading to worse health outcomes. Although this might seem to be a data issue, the choice of focusing on the average is a methodological one. Most statistical methods rely on making statements for averages of groups of individuals, which could exacerbate inequalities for minoritised groups and is something that methodologists should be aware of in their research. Researchers could consider adapting their analysis and re-weighting to gain further insights into how their methods change results for minoritised groups.

#### Dissemination, implementation and impact

Statistical methodology research is a step removed from the clinical application, which can make it challenging to communicate the methodology to diverse audiences. As discussed in the PPI section, methodologists should be able to explain their research and how it will feed into healthcare decisions. Training in this skill and inclusion of PPI is essential for effective and impactful dissemination of technical methodology research. PPI resources, including tutorials, animations, and glossaries, can improve communication with a wider and more diverse audience. This will improve the uptake of methods and encourage their application to extensive health applications.

For statistical methodology research, there is often a time lag between developing or assessing methods and their uptake. This can make the impact of statistical methodology harder to measure as they are often not valued until they are implemented in applied clinical research and then go on to change clinical decision-making. As the impact of methodological research is delayed, it can take a long time to discover when statistical methods are exacerbating existing inequalities and biases. Although it can be challenging to predict when this occurs by methodologists, assessments by diverse groups of individuals and appropriate testing of methods, as previously described, may help to identify the impacts.

Additionally, as previously discussed, medical statistics methodology research aims to improve health and researchers should also aspire to, where possible, address health inequalities in their research. This may involve viewing statistical methodology through an EDI lens, adapting research questions to ensure the research is reducing health inequalities, and clearly communicating when EDI principles have been adopted in papers and wider dissemination activities. However, an additional issue faced by methodological researchers is that the inclusion of EDI principles in papers that aim to be published in mainstream methodological journals might receive resistance from publishers for being too niche for the mainstream audience or they might not receive the same level of citations or interest. It is therefore essential that inclusion and diversity in publishing activities are adopted and appreciated more widely by methodological researchers, for example, by raising awareness and in the peer review process.

#### Budgeting for inclusion

Several additional costs need to be included in research budgets, specifically for statistical methodology research, to fully consider EDI principles. If multiple data sets are required to ensure that the research addresses EDI principles, as previously discussed, additional budget will be necessary for obtaining access to or generating data, the extra time required for the data collection and analysis. Appropriate budgets for dissemination are also essential for increasing the breadth of dissemination of statistical methodology research to ensure its accessibility, something that is often overlooked.

General guidance for budgeting for PPI is provided in the NIHR Public Contributor Payment Guidance [21]. Statistical methodology researchers planning PPI should also consider the additional time required to facilitate diverse inclusion by improving accessibility and making individuals feel comfortable discussing statistical concepts, specifically the development of PPI resources.

In summary, the budgeting to incorporate EDI principles in statistical methodology research is similar to other research areas. However, since methodology research is one step removed from the clinical applications and a technical area requiring expertise, budgeting may have to be adapted to ensure meaningful inclusion in research projects.

### Recommendations

Through the process of theming and identifying key points that emerged throughout the meeting, we propose five key recommendations for how to consider equality, equity, diversity, and inclusion in statistical methodology research which are summarised in Table 2. The first recommendation to perform assessments of the required resources for EDI inclusion in statistical methodology projects was informed by discussion of the ‘Research Team’, ‘Patient and public involvement’, ‘Data collection’, ‘Dissemination, implementation and impact’ and ‘Budgeting for inclusion’ domains. The second recommendation refers to encouraging statistical methodologists to take an EDI lens to their research with the specific aim of reducing health inequalities through methodological development; this was informed by discussion of the ‘Data analysis and presentation’ and ‘Dissemination, implementation and impact’ domains. The third recommendation highlighting the importance of evaluating all aspects of the data sets that are used within statistical methodology research was informed by the discussion of the ‘Historical context and structural inequalities’ and ‘Selection of participants, sites and samples’ domains. The fourth recommendation focusses on clearly reporting EDI evaluations of statistical methodological projects, which was informed by the ‘Selection of participants, sites and samples’ and ‘Dissemination, implementation and impact’ domains. The fifth recommendation, to incorporate EDI into wider dissemination and publishing activities, was informed by the ‘Dissemination, implementation and impact’ domain.

**Table 2:** Recommendations for how to embed equality, equity, diversity and inclusion (EDI) in statistical methodology research

1. Perform formal assessments of the required resources for maximising EDI from the outset of statistical methodology research projects, including consideration of research team, training, PPI, and appropriate budgeting.
2. Conduct prospective and retrospective context-specific evaluations of the impact of their methodological research on exacerbating or reducing inequalities.
3. Evaluate the selection of data sets, work with multiple, diverse databases, or use data sets that have undergone equality impact assessments.
4. Clearly communicate EDI assessments and limitations, including which data sets are used and their purpose, such as illustrating or comparing methods, informing simulations, or guiding clinical practice.
5. Incorporate EDI in dissemination activities and advocate for EDI principles in the peer review process.

## Discussion

This study aimed to raise awareness of EDI issues and identify gaps in the existing guidance related to statistical methodology research. It provides a narrative discussion of the issues, drawing on experiences of statistical methodology researchers and examples from the literature that were discussed during a face-to-face meeting of statistical methodologists and EDI specialists. This work included the perspectives of EDI leads, which is important to ensure EDI remains relevant and appropriate [17]. The NIHR RDS EDI toolkit was valuable for embedding and optimising EDI in statistical methodology research. While all the domains in the original NIHR RDS EDI toolkit were relevant to this context, some modifications were needed to reflect specific additional challenges of statistical methodology research.

To our knowledge, this is the first study to explore how EDI principles can be incorporated specifically in statistical methodology research in the UK. The absence of previous research highlights a significant gap in the discussion and guidance of EDI principles in this area. While no prior studies or guidance has focussed specifically on EDI in statistical methodology research, this study aligns with the work of the University of Leicester PPI-SMART group, as described in the PPI domain. Other researchers have also published narrative summaries on including PPI in quantitative research [8, 9]. Although awareness of the importance of PPI in statistical methodology research has grown, particularly as funders increasingly require it for applications, awareness of EDI in statistical methodology research is at an earlier stage as funders begin to introduce EDI statements in application processes. The findings of this study may provide a valuable foundation to begin addressing the gap by highlighting issues and providing preliminary guidance. Future studies can build upon this work by developing additional resources to aid statistical methodology researchers in incorporating EDI in their work.

One important theme that was recurrent throughout the domains of the toolkit was the challenges faced by statistical methodology researchers, particularly those working independently or in small teams, to involve individuals from diverse backgrounds throughout the research process. Statistical methodology researchers must take the time to increase the diversity of the people they involve in projects to benefit from useful perspectives and ensure that methods are developed whilst incorporating EDI principles. Interdisciplinary collaboration with other researchers and PPI with diverse characteristics early on in project timelines is recommended. Additionally, engaging students from under-represented backgrounds will improve the diversity of future statistical methodology researchers.

Changing the approach to methodological development was another consideration for researchers to incorporate EDI principles into their work. Taking an EDI lens to adapt research questions and prioritise the development of methods that can stratify across groups rather than average over populations, could change the statistical methods that are developed. Essentially, this paper encourages statistical methodology researchers to take a step back and make a thorough assessment of how and why methods are developed and whether they affect EDI principles. The approach to data was extensively discussed throughout the day during the meeting, emphasising that the boundary between methodological development and applied health research is not clear-cut. However, an observation that was made in the meeting was that whenever any data is used, even if its use is only illustrative for a new method, statistical methodology researchers should still take responsibility for ensuring that the data upholds ethical and EDI principles.

Purposive sampling was utilised to recruit participants to the face-to-face meeting based on their knowledge and experience of EDI and statistical methodology research. All participants were also recruited locally within the East Midlands. This type of sampling relies on researcher judgement in identifying relevant participants, which can introduce bias, lead to a less diverse group, and limits generalisability. We recognise that our findings only relate to the research team that attended the workshop and the group potentially misses additional experiences and views. We have included a Researcher Positioning and Reflexivity Statement in this paper to be transparent that the discussion in this paper reflects the specific perspectives and experiences of the group. Further research is needed to seek the wider views and opinions of a broader range of individuals in statistical methodology research and statisticians that will apply the methods in health topics to understand their needs. Additionally, the views of decision makers, who interpret and implement the findings of health research, are relevant to ensure that the practical implementation of including EDI principles in statistical methodology research filters through to decision making. Bringing in the voices of public contributors from diverse and underrepresented groups is also essential to ensure that populations represented in research have input into methodological decisions that will impact them.

In this study, we aimed to provide an initial exploration of EDI considerations within statistical methodology research, guided by the NIHR RDS EDI toolkit. However, a limitation of this work is that it is not a formal qualitative study including rigorous methodology, such as thematic analysis. Instead, this paper represents an initial narrative discussion based on insights and experiences shared in the meeting and examples from the literature. While this paper provides preliminary perspectives, future work utilising qualitative methods is needed to gain a deeper understanding of EDI issues in this context.

Statistical methodology research that informs health care and provision must transform its approach to integrating EDI principles, and numerous tools are available to facilitate this process. Embedding EDI principles throughout statistical methodology research will help to improve the relevance and quality of our research, increase innovation and problem-solving, better serve the public, and build public trust. We encourage statistical methodologists to consider the recommendations in this paper and strive towards equity in all aspects of their academic work.

## Data Availability

No datasets were generated or analysed during the current study.
